# Seasonal Evolution of Size-Segregated Particulate Mercury in the Atmospheric Aerosol Over Terra Nova Bay, Antarctica

**DOI:** 10.3390/molecules25173971

**Published:** 2020-08-31

**Authors:** Silvia Illuminati, Anna Annibaldi, Sébastien Bau, Claudio Scarchilli, Virginia Ciardini, Paolo Grigioni, Federico Girolametti, Flavio Vagnoni, Giuseppe Scarponi, Cristina Truzzi

**Affiliations:** 1 Dipartimento di Scienze della Vita e dell’Ambiente, Università Politecnica delle Marche, Via Brecce Bianche, 60131 Ancona, Italy; f.girolametti@pm.univpm.it (F.G.); f.vagnoni@univpm.it (F.V.); g.scarponi@univpm.it (G.S.); c.truzzi@univpm.it (C.T.); 2 Laboratory of Aerosol Metrology, Institut National de Recherche et de Sécurité (INRS), Rue du Morvan, CS 60027, 54519 Vandoeuvre, France; sebastien.bau@inrs.fr; 3 Laboratory of Observations and Measures for The Environment and Climate, ENEA, Via Anguillarese 301, Santa Maria di Galeria, 00123 Rome, Italy; claudio.scarchilli@enea.it (C.S.); virginia.ciardini@enea.it (V.C.); paolo.grigioni@enea.it (P.G.)

**Keywords:** particulate mercury, mercury size-resolved distribution, dry deposition, meteorological parameter, backward trajectories, Antarctica

## Abstract

Size-fractionated particulate mercury (PHg) measurements were performed from November 2017 to January 2018 at Terra Nova Bay (Antarctica) for the first time. Samples were collected every 10 days by a six-stage high-volume cascade impactor with size classes between 10 μm and 0.49 μm. Total PHg concentrations were maxima (87 ± 8 pg m^−3^) in November, then decreased to values ~40% lower and remained almost constant until the end of the sampling period (~30 pg m^−3^). The trimodal aerosol mass distribution reveals that from 30% to 90% of the total PHg came in the size > 1.0 μm. Hg in the two coarse fractions was probably produced by the adsorption of oxidized Hg species transported by air masses from the Antarctic plateau or produced locally by sea ice edges. PHg in accumulation mode seemed to be related to gas–particle partitioning with sea salt aerosol. Finally, average dry deposition fluxes of PHg were calculated to be 0.36 ± 0.21 ng m^−2^ d^−1^ in the accumulation mode, 47 ± 44 ng m^−2^ d^−1^ in the first coarse mode, and 37 ± 31 ng m^−2^ d^−1^ in the second coarse mode. The present work contributed to the comprehension of the Hg biogeochemical cycle, but further research studies are needed.

## 1. Introduction

Mercury (Hg) is an element naturally occurring in the Earth. This metal continues to be of high concern due to its intrinsic characteristic, e.g., volatility, mobility, persistence, and strong tendency to bioaccumulate in food chains. Mercury has been considered as a Priority Pollutant by the United States Environmental Protection Agency (US-EPA) since 1977 and it is addressed under the Heavy Metals Protocol to the Convention on Long-range Transboundary Air Pollution that entered in force in 2003.

The atmosphere plays a key role in mercury transformation, removal, and transport to various ecosystems, even to remote areas through long-range processes [1,2,3]. Sources of mercury in the atmosphere are both natural (volcanoes, forest fire, geothermal vents, evaporation from soil and waters) and anthropogenic (fossil fuel combustion, mining and extraction of minerals, production of non-ferrous metals, cement production, artisanal and small-scale gold mining, dental applications, batteries), with the latter having contributed for the most part [4,5,6,7].

The United Nations Environment Programme (UNEP) published a new global inventory of mercury emissions to air in 2018 [8]. In 2015, about 2220 tons of mercury were emitted in the air from 17 key sectors worldwide. These emissions are 20% higher than those estimated for 2010. In fact, the remedial actions applied by North America and the European Union have resulted in modest decreases in emissions, probably because of the increase of economic activity (especially in Asia) and the use of mercury-added products that have offset any efforts to reduce mercury emissions.

Obviously, Asiatic regions (primarily East and Southeast Asia) are the largest emitters of mercury worldwide, contributing about 49% of the global emissions, followed by South America (18%) and Sub-Saharan Africa (16%) [8]. 

The chemistry of atmospheric mercury is quite complex. Typically, three forms of Hg can be recognized in the ambient air: the gaseous elemental mercury (Hg^0^), the gaseous oxidized mercury (Hg^2^), and the particulate mercury (PHg). Hg^0^ is the main species of Hg in the atmosphere (>90% of the total atmospheric mercury [9] with residence time of about 1 year [10]); thus, it can be transported globally, even to remote areas [11,12]. The fate of Hg^0^ is to be oxidized into the more reactive and water-soluble gaseous Hg^2^, and/or into the particulate matter (PHg [13]). Due to its high dry deposition velocities and scavenging coefficients [14], PHg is more important than Hg^0^ with respect to atmospheric deposition, and thus to the transport of mercury from the atmosphere to the terrestrial and aquatic ecosystems [15]. Once deposited, Hg can be methylated to methylmercury in aquatic systems, leading to bioaccumulation and toxic effects in biota and humans [16,17].

Far from the civilized world, at least 3000 km, Antarctica is considered the last pristine environment of the Earth. Nevertheless, it is not escaping the impact of anthropogenic emissions of several pollutants that reaches Antarctica by long-range transport processes. 

Several studies on atmospheric Hg highlighted the role of the Southern Hemisphere emissions on the mercury input control in Antarctica with respect to the limited contributions by local active volcanoes, geothermal events, and human activities on the continent [3,18]. The Hg cycle has unique features in Antarctica and more in general in polar regions with respect to the lower-latitude areas [19]. The main phenomena that characterize polar regions are the so-called atmospheric mercury depletion events (AMDEs). During springtime, gaseous elemental Hg is depleted from the lower troposphere, since it is oxidized by halogen radicals, and deposited on the ground or bound to atmospheric particles more rapidly than anywhere else [3]. This discovery, made in Alert (Canada) by Schroeder et al. [10], has given a considerable boost to the research on atmospheric chemistry and on the Hg biogeochemical cycle. A series of following research studies also reported several Hg depletion events in Antarctica near some coastal Antarctic stations, e.g., Neumayer [20], Mario Zucchelli [21], Dumont d’Urville [22], and McMurdo [23]. In the last decades, many studies focused on the measurements of tropospheric Hg^0^ concentrations in Antarctica and on the mechanism behind the AMDEs during the Antarctic springtime.

Despite to its key role in the global mercury biogeochemical cycle, PHg studies are very sporadic with respect to Hg^0^ and Hg^2^ and very little is known about the size distribution of PHg in the atmosphere. PHg measurements were carried out in few Antarctic areas, e.g., at Mario Zucchelli Station [24]; at the German Neumayer station [25]; at McMurdo Station [23]; and at the South Pole [26]. Not one of these studies, which date back more than 10 years, has considered the PHg size distribution. However, particle diameter is a key factor to estimate the Hg depositional fluxes on the ecosystems, since it affects aerodynamic resistance, atmospheric residence time, gravitational settling, and long-range transport [27,28]. Particle size distribution, moreover, provides valuable information on the potential sources of PHg [7]. Several studies carried out around the world have showed a possible marine source for PHg coarse fraction in coastal and marine areas, while in urban and industrialized regions, PHg in fine particles showed an anthropogenic input [29,30].

The study of mercury in Antarctica is challenging due to the extreme operative conditions; nevertheless, much efforts is necessary in order to gain more insight in some unclear environmental aspects, e.g., the role of the Antarctic continent in the global biogeochemical cycle of Hg and the mechanisms characterizing the deposition of Hg to the coastal ecosystems [3].

**Figure 1:**
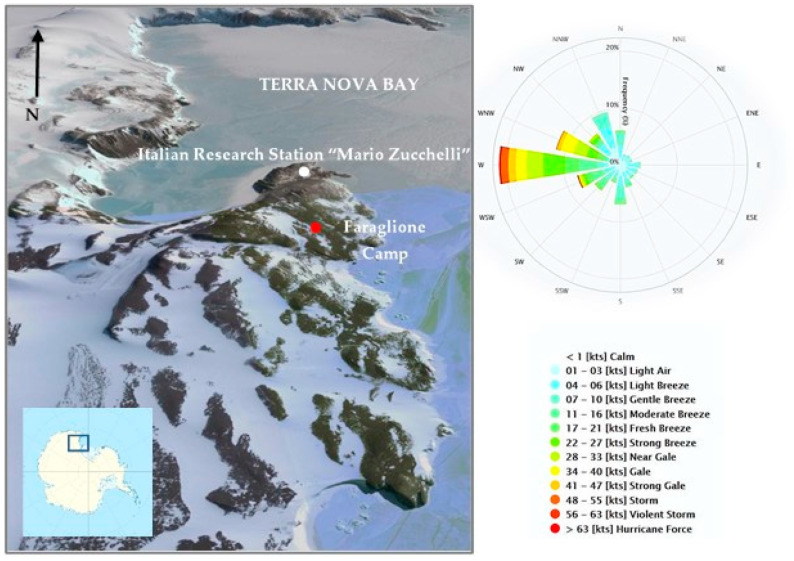
Map of the sampling site (Faraglione Camp) and the Italian Mario Zucchelli Station. Wind rose plot with the prevailing wind direction over the sampling site is also reported.

Under the framework of the Italian Antarctic Programme, the determination of atmospheric particulate mercury at Terra Nova Bay (Victoria Land, Antarctica, Figure 1) was carried out. To our knowledge, this study is the first to measure the size distribution of atmospheric PHg in Antarctica during the austral summer. Our principal goals were to: (1) measure the PHg concentrations in one of the remote areas of the world; (2) evaluate the PHg distribution between different size fractions of PM10; (3) characterize the summer evolution of the size-resolved PHg fractions; (4) investigate the relationship between PHg and meteorological parameters; (5) estimate the dry deposition fluxes of size-resolved particulate mercury; and (6) assess the potential sources of PHg at Terra Nova Bay and the possible impact of long-range transport. 

## 2. Results and Discussion

### 2.1. PHg Concentrations in Antarctica

Table 1 shows a summary of the PHg concentrations in PM10 obtained as sum of the size-segregated fractions, and the corresponding data of the atmospheric concentrations referred both to actual (mean temperature, 268 K and mean pressure, 954 hPa) and standard air (298 K, 1013 hPa) during the Antarctic summer 2017–2018.

First, it can be noted that the correction computed for standard air is negligible (differences in the range between 2% and 4%) if compared with the precision in the volume sampled (±10%). By consequence, in the following paragraphs, results referred to actual air will be discussed.

PHg atmospheric concentrations measured at Faraglione Camp during the austral summer showed the following values, given as means (interquartile range): Dp, <0.49 μm, 1.3 (0.61–1.6) pg m^−3^; 0.49−0.95 μm, 3.5 (3.0−3.9) pg m^−3^; 0.95−1.5 μm, 11 (4.6−14) pg m^−3^; 1.5−3.0 μm, 18 (6.8−29) pg m^−3^; 3.0−7.2 μm, 18 (11−20) pg m^−3^; 7.2–10 μm, 5.0 (3.1−5.5) pg m^−3^. 

On average, the total PHg content in PM10 was 51 ± 27 pg m^−3^ with a range of 30–90 pg m^−3^.

The PHg concentrations obtained in the present work were compared to the literature data available from the same and/or other Antarctic regions and from other areas worldwide (Table 2). 

As already stated, the measurements of Hg in Antarctica are very sporadic. Although there is great variability, a general agreement with data previously reported can be recognized. Our data were slightly higher, even if in the same in order of magnitude, than those reported by Sprovieri et al. [10] in the same Antarctic site. 

Compared globally, our values confirmed what was previously discovered by studying atmospheric mercury in Antarctica, i.e., surprisingly higher concentrations of PHg (from ~4 pg m^−3^ to ~660 pg m^−3^) with respect to those measured in urban and industrialized areas [32]. These high concentrations of oxidized mercury species could be the consequence of phenomena occurring in Antarctica (e.g., AMDEs) and making the Hg biogeochemical cycle unique in the world [20,23]. Measurements of Hg in Antarctic coastal areas suggested that the production of Hg oxidized species (i.e., reactive gaseous mercury, RGM, and/or PHg) seemed to be related to gas-phase oxidative processes activated not only by AMDEs during springtime, but also by potential oxidants (^·^OH, ${\mathrm{HO}}_{2}^{\cdot }$, $O^{\cdot }$, ${\mathrm{NO}}_{3}^{\cdot }$) present in air masses originating from the Antarctic plateau and produced by photochemical reduction processes in the snowpack [25]. These air masses enriched in oxidants and oxidized Hg species reached the coastal areas through katabatic winds [22]. At Terra Nova Bay, Sprovieri et al. [21] also measured very high concentrations of reactive gaseous mercury (RGM, Hg^2+^) in the absence of Hg^0^ depletion events. This Hg species tended to be dry deposited and/or rapidly scavenged by aerosol particles [24,41]. The authors suggested a local gas-phase oxidation of Hg^0^ promoted by H_2_O_2_ that shows high concentrations in the remote and clean marine boundary layer (MBL) of Antarctica. Thus, MBL is a highly oxidant environment in polar regions where H_2_O_2_ reacts with the HONO released from the snowpack producing OH and NO_2_ [42].

Finally, influences from the recent enhanced anthropogenic emissions in the Southern Hemisphere, especially because of the widespread small-scale gold production [18], cannot be excluded. 

### 2.2. Size-Resolved Mercury Distribution

To gain more information on the possible sources of mercury at Terra Nova Bay, the size-resolved PHg distribution was performed. Cascade impactors with various cut-off diameters, flow rates, and sample substrates were usually employed to study the size distribution of several constituents in the ambient air [43]. The main issues of cascade impactors as tools to study the size distribution are inherent to some physical phenomena or artifacts that can affect particle collection. In fact, particles may bounce, adhere, blow off when ending up on a lower stage, or deposit in the passageways between stages. These result in possible overlap between one impactor stage and the subsequent stage [29,43]. To overcome these issues, an inversion procedure developed by Bau et al. [43] (see below in Section 2.5) was applied to the whole dataset. This allowed to convert the raw cascade impactor data to continuous size distribution. 

**Figure 2:**
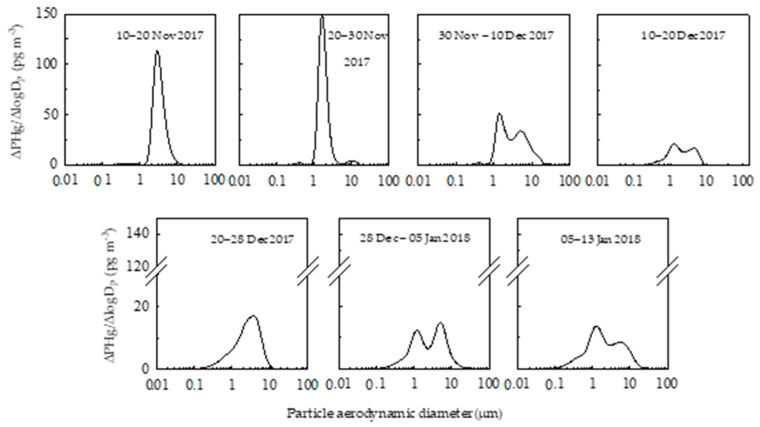
Size distributions of PHg in the different size-segregated aerosol fractions collected at Faraglione Camp (Terra Nova Bay, Antarctica) during summer 2017–2018.

During the austral summer 2017–2018, a trimodal distribution of PHg for each sample can be recognized, except for two samples (10–20 Nov 2017 and 20–28 Dec 2017) (Figure 2). The three “peaks” or modes obtained can be ascribable to: an accumulation mode, PHg_0.1–1.0_ (0.1 µm < D_p_ < 1.0 µm); a first coarse mode, PHg_1.0–2.5_ (1.0 µm < D_p_ < 2.5 µm), and a second coarse mode, PHg_2.5–10_ (2.5 µm < D_p_ < 10 µm).The PHg concentrations in the accumulation and in two coarse fractions showed the following results, given as mean (interquartile range): PHg_0.1–1.0_, 4.1 (2.4–5.6) pg m^−3^, PHg_1.0–2.5_, 39 (10–63) pg m^−3^, PHg_2.5–10_, 15(11–14) pg m^−3^. The highest percentages of PHg were found in the first coarse fraction PHg_1.0−2.5_ accounting from ~30% to ~99% of the total particulate mercury. The other two PHg fractions, PHg_0.1–1.0_ and PHg_2.5–10_ contributed to a less extent to the total mercury content; the percentages varying from ~3% to ~30% and from ~20% to ~40%, respectively. 

In general, studies on size-resolved PHg evolutions are very limited all over the world. When considering the PHg distribution in the PM10 in various urban, industrialized and rural areas disparate results were obtained. Several studies [40,44,45,46] carried out in areas with different anthropogenic inputs (vehicular traffic, industrial activities, metal mining) observed a bimodal distribution of PHg in the atmospheric aerosol. They recognized a fine mode (with size in the range 0.5–1.0 µm) and a coarse mode, the size range of the latter changing on a case-by-case basis (from 1.0–2.5 µm to 4.0–6.0 µm, to 2.5–10 µm). Kim et al. [29], studying size-segregated PHg in two Korean cities, found a unimodal distribution with a significant seasonal difference in the dominant size. During winter, the fine mode with a peak in the 0.32–0.56 µm size range was dominant, while in summer, the coarse fraction (1.0–2.5 μm size range) became more important. Li et al. [7] found a mixed situation in Beijing (China): PHg showed a unimodal distribution in winter with a mode in the 0.56–1.0 µm size range, a bimodal distribution in summer (two modes in the size ranges 0.56–1.0 µm and 2.5–5.6 µm, respectively), and a multimodal distribution during spring. Han et al. [39] measured a trimodal distribution of PHg in the ambient air with three principal fractions: a nucleation mode (<0.05 µm), an accumulation mode (0.05–2.0 µm), and a coarse mode (>2.0 µm).

Generally, all the studies carried out on PHg size distribution highlighted the significant contribution of the fraction with the 1.0–2.5 µm size range to the total atmospheric particulate content.

### 2.3. Seasonal Evolution of PHg in Antarctica

**Figure 3:**
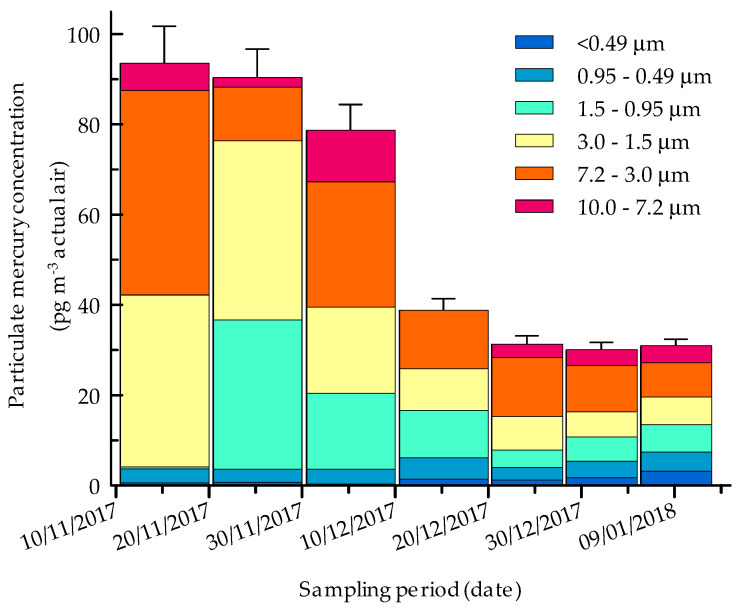
Seasonal trend of size-segregated particulate mercury concentrations at Faraglione Camp from November 2017 to January 2018. Error bars are referred to SD of the different PHg fraction sums, calculated as the square root of the sum of variances.

Figure 3 shows the variation of PHg during the austral summer. Total PHg concentrations were maxima (87 ± 8 pg m^−3^) and almost constant during November. At the beginning of December, PHg decreased to values ~40% lower than the spring and then remained almost constant until the end of the sampling period (~30 pg m^−3^).

PHg maxima during summer season were recorded in several coastal and inland Antarctic stations, as a consequence of both AMDEs and gas-phase oxidation of Hg^0^ promoted by the strong oxidants ^·^OH, ${\mathrm{HO}}_{2}^{\cdot }$, $O^{\cdot }\;$ or ${\mathrm{NO}}_{3}^{\cdot }$ [21,22,23,25,42,47]. 

**Figure 4:**
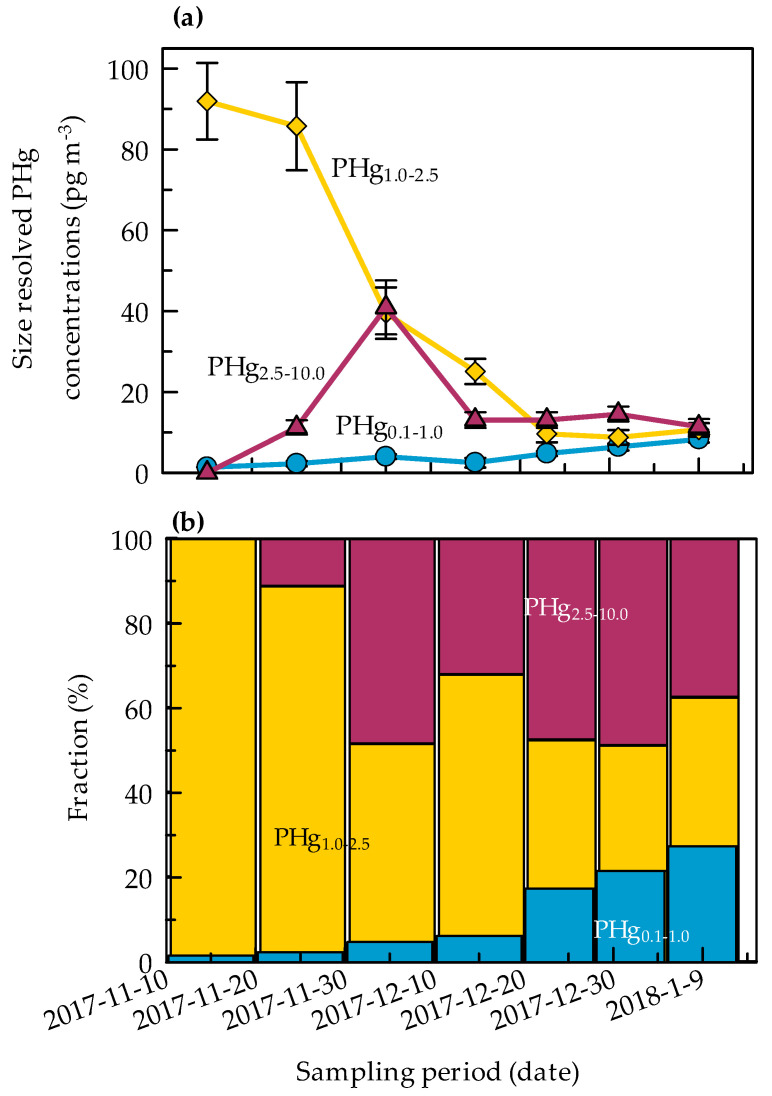
Seasonal evolution of different PHg modes (PHg_0.1–1.0_; PHg_1.0–2.5_ and PHg_2.5–10.0_) (**a**) and their relative contribution to the total mass concentration (**b**) over the sampling period. Error bars ± SD (calculated as the square root of the sum of variances).

The seasonal trend and the relative contribution of the three modes to the total particulate mercury during the austral summer are reported in Figure 4. It can be noted that PHg_1.0–2.5_ (even if with a more pronounced decrease than total mercury) is responsible of the total PHg evolution during summer, as it is also confirmed by correlation matrix (see below in Table 3). PHg_1.0–2.5_, in fact, represented a significant fraction of the total PHg, varying from ~90% at the beginning of the season to ~30% at the end of the sampling campaign. PHg in the coarsest fraction (PHg_2.5–10_), showed a maximum in mid-December, but rapidly decreased to constant values (~40% of the total PHg) until the end of season. On the contrary, PHg_0.1–1.0_ counted for a very small fraction of the total PHg (from ~1% to ~30%) and gradually increased during the sampling campaign to reach the highest values in mid-January.

These distributions demonstrated that PHg could have different formation mechanisms. Besides the direct emissions from natural or anthropogenic sources, Xiu et al., 2005 [13] suggested two main formation mechanisms as secondary sources of PHg: adsorption of gaseous mercury on particles and chemical gas–particle transformation. Generally, PHg in coarse fractions forms through adsorption of gaseous mercury onto coarse particles generated from natural sources, such as sea salt aerosols and dust, and mechanical processes from anthropogenic sources [29]. On the contrary, gas–particle transformation plays a key role in the PHg formation in fine particles [13].

The atmospheric particulate matter (PM) in Terra Nova Bay for the austral summer showed a trimodal size distribution, with an accumulation mode in 0.1–1.0 µm, and two coarse modes, one in the range 1.0–3.0 µm and the second one in 3.0–10.0 µm. The accumulation mode accounted from ~50% to ~90% of PM in Antarctica (the two coarse modes representing from ~5% to ~35% of PM) and increased in mid-December at the same time of the pack-ice reduction [48,49]. As reported in literature [49,50] the sea spray contribution (Na^+^, Mg^2+^, Cl^−^ and sea salt ${\mathrm{SO}}_{4}^{2-}$) dominates the coarse fraction, whereas methanesulfonate and not-sea-salt ${\mathrm{SO}}_{4}^{2-}$ prevail in the fine particles, showing maxima during summer, as they originate from the oxidation of dimethylsulfide emitted in the atmosphere by the phytoplankton blooms.

In general, if more particles from a specific PM fraction are present in the atmosphere in some period, we can expect that there is also more fraction-bound Hg in that area. Nevertheless, the PHg size distribution in the analyzed period did not occur in the diameter range defining the most abundant PM fraction. This different behavior confirms PHg production mechanisms other than the only adsorption process on particles, e.g., the chemical gas–particle transformation. 

Several studies indicate that the Southern Ocean is a net source of gaseous mercury in the Antarctic summer, especially when the pack ice melts, contributing more than half of the atmospheric concentration [42,51]. Concurrently, a large amount of sea salt aerosol is released in the atmosphere during the season, participating to adsorption and gas–particle partitioning events with the newly emitted gaseous mercury [29]. 

**Figure 5:**
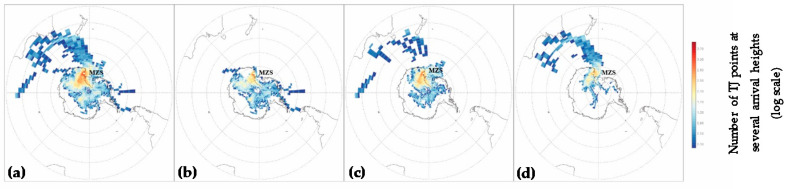
Total number of points of TJ, arrriving at 500, 1000, 1500, and 2000 m above MZS, laying within each area of 4° × 1° (filled polygons). Panel (**a**) refers to the whole TJ’s dataset while the (**b**–**d**) highlighted the differences between November, December, and January, respectively. Data are reported in a logarithmic scale.

This marine contribution could only partially affect the PHg distribution recorded in our work. In fact, in the 2017–2018 Antarctic summer, the pack ice melted at the end of the sampling campaign. Nevertheless, wide areas of the Ross Sea were ice-free in January, contributing to the evasion of great amount of marine aerosol, which can reach the study site. This is supported by the HYSPLIT model simulations showing air masses originating from the Southern Ocean or Ross Sea ice-free coastal areas reaching Faraglione Camp (Figure 5d). Trying to investigate the long-range transport from anthropized regions, we imposed an additional threshold considering only back-trajectories (TJ) points, between 100 and 1000 m, and also below 48°S for almost 5% of the time (6 h). Results (not shown) confirm the passage above the Tasmanian Sea closed to the Australian coasts. Selected TJ points below 48° S were prevalently related to few events at the beginning of January when PHg_0.1–1.0_ concentrations increased. We hypothesized that PHg_0.1–1.0_ increasing could be explained in terms of gas–particle partitioning of both Hg^0^ and Hg^2+^ with the methanesulfonate and the not-sea-salt sulphate released from the sea surface. Based on the HYSPLIT models, a contribution of the long-range transport of fine particles from anthropized areas to the PHg size distribution at Terra Nova Bay cannot be excluded (Figure 5d). Nevertheless, further studies are necessaries in order to confirm or refute this hypothesis.

In November, when the pack ice covered large areas of the Ross Sea, the high levels of PHg in the coarse fraction were probably due to other sources than the marine one. Sprovieri et al. [21] suggested a local production of oxidized mercury species by the presence of sea ice edges during the polar summer. Angot et al. [22] suggested the advection by katabatic winds of inland air masses from the Antarctic plateau to coastal areas. These air masses are enriched in oxidants and oxidized Hg species produced by reactions occurring within the shallow boundary layer on the Antarctic plateau [25] and cause the high Hg^2+^ species concentrations and elevated levels of total Hg in surface snow samples recorded at the French station Dumont d’Urville (DDU) during summer. 

**Figure 6:**
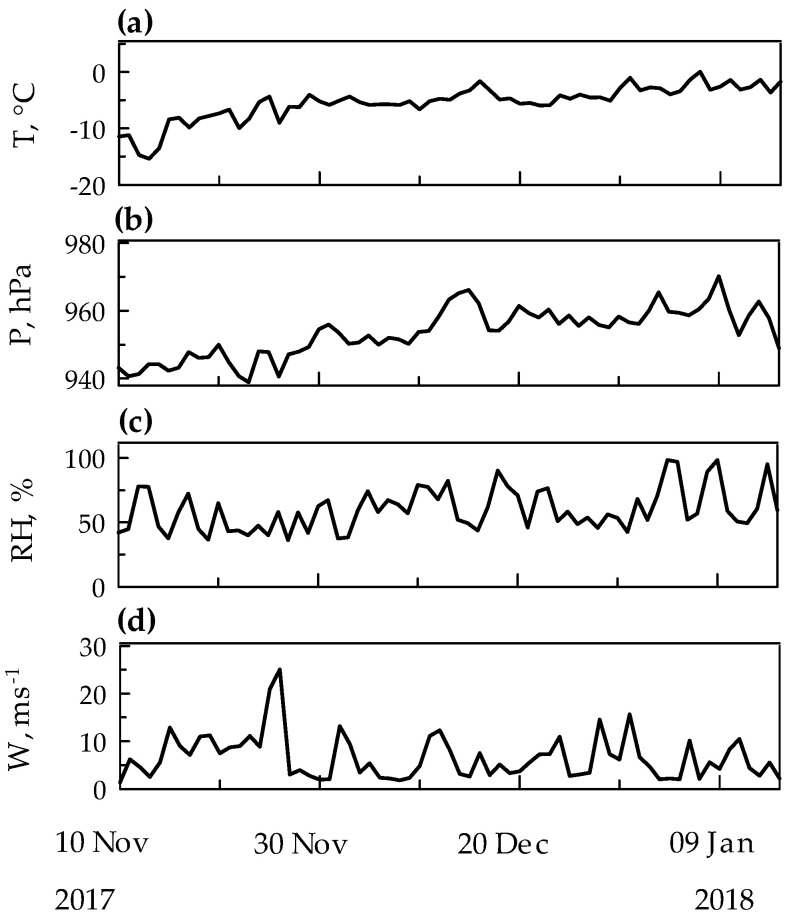
Meteorological parameter variations at Faraglione Camp during the sampling period. The panels (**a**–**d**) show the average day to day variation of air temperature (T, °C), relative humidity (RH, %), pressure (P, hPa) and wind speed (W, m s^−1^), respectively.

As highlighted by the HYSPLIT model, until January, Terra Nova Bay was affected by air masses originating from the Antarctic plateau (Figure 5b,c) reaching the sampling site, sometimes at very high speeds (see further in Figure 6a). We hypothesized that the high concentrations of PHg in November could be produced by the adsorption of oxidized Hg species on coarse particles, as these Hg species were either transported by air masses from the Antarctic plateau or produced locally by sea ice. 

### 2.4. PHg and Meteorological Conditions

Several studies carried out in several areas worldwide have highlighted the possible influence of meteorological conditions on the temporal variation of PHg [7,24,29,52]. In this study, we examined the relationships between PHg concentrations in the three size fractions and meteorological parameters, such as temperature, atmospheric pressure, relative humidity, and wind speed. Therefore, the correlation matrix with Pearson’s coefficients was calculated, and statistical results are summarized in Table 3. 

The correlation matrix highlights the significant contribution of PHg_1.0–2.5_ to the total PHg evolution during summer, as previously noted. PHg_1.0–2.5_ was also negatively correlated with PHg_0.1–1.0_, and both these two fractions showed an opposite relationship with temperature and surface pressure. 

Generally, the negative correlation of PHg with temperature highlighted the deposition of mercury on particles as main process of PHg formation. By contrast, the positive link of PHg to temperature revealed photochemical transformation as key pathway formation of this fraction [29,53]. In the present work, the opposite behavior of the two PHg fractions with temperature could be explained as a seasonality effect. The temperature rising typical of summer season in association with moderately calm winds promoted the gas-partitioning of PHg onto fine particles emitted by sea spray, contributing to the increase of PHg_0.1–1.0_. The low temperature at the beginning of season promoted the oxidized gaseous mercury species, reaching the study area through katabatic winds, to be adsorbed onto the coarse particle surfaces [24,54]. 

RH seemed to have no influences on PHg distribution. Nevertheless, a quite high, even if not significant, negative correlation with PHg_1.0–2.5_ can be observed. From mid-December, a change in the two coarse fractions contribution to total PHg occurred, the latter remaining practically constant during the same period (excepting for a slightly increase in mid-January, as result of the PHg_0.1–1.0_ rising). It could be possible that a water condensation on the first coarse mode particles occurred, causing them to grow in size [55] as RH slightly rise during summer. A similar event was reported in our previous work on PM size distribution [48], when a shift of the 1.0–3.0 size mode in the range of 3.0–10.0 µm was observed as RH increased during the summer 2014–2015. Further studies are necessary in order to verify this previous hypothesis. 

The other meteorological parameter strongly influencing the seasonal behavior of atmospheric Hg is the wind [56]. As shown in Table 3, however, the correlation between median 10-day wind speed and all the three PHg fractions was very low and not statistically significant. This lack of significant correlations between wind and PHg concentrations suggests that influences from this parameter were not directly related to atmospheric PHg [57]. However, deposition phenomena could occur when the wind calmed down, explaining the PHg decrease in December. 

### 2.5. Estimation of PHg Dry Deposition Fluxes

Besides the scavenging of atmospheric particulate mercury by precipitation (e.g., snowfall), PHg can also be removed from the atmosphere and deposited into terrestrial and aquatic ecosystems by physical processes (e.g., atmospheric diffusion, gravity settling), the so-called dry deposition. PHg dry deposition is crucial to deepen our insight into the mercury biogeochemical cycle, especially in Antarctica, and to predict the impact of deposition phenomena on fragile ecosystems. However, despite the key role of these processes, very few studies were carried out on the size resolved PHg deposition [58]. 

Dry deposition fluxes of PHg were calculated using size-fractionated deposition velocity as computed by the global multi-scale Chemistry and Transport Model MOCAGE [59] (see below in Section 3.6 [59]) and corresponding ambient concentrations of PHg measured in this study. PHg dry deposition fluxes of total and size fractions during the Antarctic summer at Faraglione Camp are shown in Table 4. 

The dry deposition fluxes weighed over the 10-day sampling period for accumulation, first and second coarse modes were (results given as mean ± SD) 0.36 ± 0.21 ng m^−2^ d^−1^, 47 ± 44 ng m^−2^ d^−1^, and 37 ± 31 ng m^−2^ d^−1^, respectively. Results highlighted that the seasonal variation of size-resolved deposition fluxes reflected the pattern in the seasonal variation of PHg concentrations in the three size fractions. The relative contribution of the size-resolved deposition fluxes to the total showed that more than 90% of the PHg dry deposition flux in summer came from particles larger than 1.0 µm. PHg_0.1–1.0_ dry flux was very low, contributing for less than 1% to the total season flux. Although PHg was mainly present in the size range 1.0–2.5 μm, the PHg in the size range 2.5–10 μm contributed ~67% to the total dry deposition in summer. Dry deposition velocities are strongly size-dependent and increase with particle size. Therefore, dry deposition of PHg in sizes between 2.5 and 10.0 μm occupied more than 60% of the total deposition flux.

To our knowledge, there are no measurements of particulate mercury dry deposition in Antarctica. Angot et al. [22] measured a Hg^0^ dry deposition velocity of 9.3 × 10^−5^ cm s^−1^ in winter at Dome C. The authors also reported the total (wet + dry) deposition fluxes in three Antarctic stations (Troll, TR; Dome C, DC and Dumont d’Urville, DDU) for the year 2013 obtained through multi-model simulations (ECHMERIT; GEM-MACH-Hg, the mercury version of the Global Environmental Multi-scale; Modelling air quality and Chemistry model GEOS-Chem; and GLEMOS, the Global EMEP Multi-media Modelling System): 1.0, 3.3, 2.5, and 3.9 μg m^−2^ yr^−1^ at TR, 0.8, 1.5, 0.8, and 1.1 μg m^−2^ yr^−1^ at Dome C, and 4.3, 9.7, 9.7, and 4.1 μg m^−2^ y r^−1^ at DDU according to GLEMOS, GEOSChem, GEM-MACH-Hg, and ECHMERIT, respectively.

Since summer deposition accounted for a high proportion (50–80%) of the total annual flux in coastal areas [22], we can greatly approximate our results to annual basis. Year-round total PHg flux was ~3.0 µg m^−2^ y^−1^, quite similar to that predicted by the multi-model simulations in Angot et al. [22]. It is worth noting that wet mercury deposition (which is the principal mechanism of PHg scavenging from the atmosphere) was not considered in our rough approximation. Further studies are necessaries in order to estimate total mercury fluxes in Antarctica and to assess the seasonality effect on mercury deposition.

The PHg dry deposition fluxes of the present work were slightly higher than those estimated in some remote areas (e.g., Tibetan plateau [57]; Appledore Island [52]), but one or more order of magnitude lower than those recorded in polluted urban and suburban areas, such as Beijing [60]; Taiwan [45]; northern China [15]; some areas in Central Europe, Northern Europe and North America [61,62].

Due to limited knowledge on particle dry deposition, the used parameterization with empirical and simplified formula could over or under-estimate the PHg dry deposition fluxes. Therefore, further studies are necessaries to evaluate the dry deposition fluxes of particulate mercury in polar regions.

## 3. Materials and Methods 

### 3.1. The Study Area

Aerosol samples were collected at Faraglione Camp (74.7161° S–164.1150° E), about 3 km southern from the “Mario Zucchelli” Italian Station (MZS). MZS is a summer-based station, being operative from mid-October to the beginning of February. Consequently, research field activities can be performed only from November to the end of January. Details of the site have been reported elsewhere [48,63,64,65]. A map of the Mario Zucchelli Station area, the Faraglione Camp and the volcano Melbourne is reported in Figure 1.

The Automatic Weather Station of the PNRA Meteo-Climatological Observatory [66] recorded the values of the principal meteorological parameters (air temperature, relative humidity, ambient pressure, wind speed and direction) close to the study area [67]. During the reference period, results were reported as mean (minima–maxima): temperature, −6.2 °C (−15 °C–0 °C), pressure, 953 hPa (939 hPa–970 hPa), relative humidity, 62% (36%–98%). A general increase of air temperature and pressure can be observed during summer (Figure 6a,b). The relative humidity showed a less pronounced variation, with the lowest values in November with respect to the rest of summer (Figure 6c).

The first part of summer was characterized by strong surface winds, reaching wind speeds of ~25 m s^−1^ (~50 knots, Figure 6d). In fact, Terra Nova Bay is one of the Antarctic “confluence zones” between the Reeves and Priestly glaciers, and it is characterized by intense katabatic winds responsible for the formation of a polynya (open water area) which persists also through winter [68]. In December and January, winds were low (~5 m s^−1^) with occasionally some peaks at 10–15 m s^−1^ (~20–30 knots). 

Due to the strong katabatic effects, the most frequent surface wind direction was West (~30%) or West-Northwest (~20%) (see the wind rose plot in Figure 1).

### 3.2. Field Sampling

The aerosol samples were collected at Faraglione Camp from November 2017 to January 2018. Measurements were made by five-stage high-volume cascade impactor (Tisch-Environmental, mod. TE-235, Analytica Strumenti, Pesaro, Italy; see Annibaldi et al. [69,70] for further details) in combination with a 10-μm pre-separator for the effective collection of six sub 10 μm fractions (we can therefore consider using a six-stage cascade impactor). The 50% cutoff aerodynamic diameters of the different stages are stated by the manufacturer at 10 μm, 7.2 μm, 3.0 μm, 1.5 μm, 0.95 μm, and 0.49 μm. Particles with aerodynamic diameter smaller than 0.49 μm are collected on backup filter. The flow rate for the cascade impactor was 1.13 m^3^ min^−1^ (±10%). A sampling strategy of 10 days was adopted to comply with the requirements of trace element determination [71], excepting for the last two samples for which sampling period of eight days was applied.

Before aerosol collection, the sampler was cleaned inside and outside by repeated washings using ultrapure water (Milli-Q, Millipore, Bedford, MA). The impactor plates were covered with slotted 6’’ × 7’’ cellulose filters, type Tisch TE-230 WH, the backup filter with an 8’’ × 10’’ borosilicate glass filters with a tetrafluoroethylene (TFE) coat, type Pallflex^®^ T60A20 (Pall Lifesciences Corp, Washington, NY, USA). Both cellulose and TFE filters were decontaminated following a procedure previously set-up [70].

To check the background contamination, blank filters (“field blanks” and “blanks as received”) were also collected in the field approximately once every month. These were simply installed onto the switched-off samplers for a few tens of minutes and then treated as the sample filters.

Samples and field blanks were stored in decontaminated Petri dishes (for decontamination procedure see Annibaldi et al. [70]) at ‒20 °C until analysis.

### 3.3. Mercury Analysis

Mercury concentrations from each sample (membrane filters) were directly analyzed by using a Direct Mercury Analyzer^®^ (DMA-80, Milestone, Italy). A detailed description of the DMA-80 was given in the study of Melendez-Perez et al. [72]. Generally, ~30 mg of each filter sample was loaded directly into the DMA and analyzed using the following procedure. First, each sample was placed in a sampling boat and then transferred to a combustion tube containing the catalyst. The sample was thermally decomposed at 650 °C in an oxygen atmosphere for 3 min and 30 s after the drying at 200 °C. Hg vapor was collected in a gold amalgamator that was heated to 650 °C for 3 min. The released Hg was transported to a heated cuvette at 125 °C, and then analyzed by atomic absorption spectrometry (AAS) equipped with a silicon UV/Vis spectrophotometer diode detector. 

### 3.4. Quality Control

Accuracy of the measurements carried out in our laboratory is usually checked by the analysis of various certified reference materials (CRMs) [71,73,74,75,76]. Since no certified reference material was available for PHg determination in atmospheric particulate matter, the accuracy of the measurements was checked by computing the percentage of recovery by triplicate analyses of filters spiked with Hg standard solutions in two Hg(II) mass levels (typically, 0.30 ng and 1.0 ng). Results of the recovery test showed the following percentages: 83 ± 2% for the filters spiked with Hg-0.30 ng, 97 ± 4% for those spiked with Hg-1.0 ng.

The repeated determinations of PHg in the different PM10 fractions gave repeatability (expressed as RSD%) of ± 6%.

The detection limit of the direct Hg analyzer was 0.018 ng (range, 0.018–0.025 ng). 

The field blanks and the blanks as received were analyzed in the same manner as environmental samples. Blank values corresponded, on average, to 6% of sample values (*n* = 6). The obtained values for blanks were subtracted from the PHg concentration measured in each sample. 

### 3.5. Data Inversion and Statistical Analysis

The mass size distribution of the atmospheric PHg concentrations averaged over the sampling period was performed by applying an inversion methodology described by Bau and Witschger [43].

This procedure bases on a deterministic method firstly set up by Twomey, 1975, and further improved by different authors [43]. Detailed description of the inversion procedure applied to the raw data obtained by our cascade-impactor is given in Illuminati et al. [48]. 

Statistical analysis was carried out using Statistica package (StatSoft; vers. 8.0). For all tests, the *p* value of < 0.05 was considered as statistically significant.

### 3.6. Calculation of The Hg Dry Deposition Flux

PHg dry deposition fluxes were calculated by the following equation [60]:

(1) $$F=\;\sum^{\;}C_{\mathrm{PHg}}\times V_{d}$$

where *F* is the dry deposition flux of PHg, *C*_PHg_ is the concentration of PHg in each size mode, and *V*_d_ is the corresponding dry deposition velocity.

As well known, dry deposition velocity was influenced by several factors (meteorological conditions, receptor surface characteristic, and particle size [61]. In this study, a size-resolved particle dry deposition model developed by Nho-Kim et al. [59] were used to estimate the dry deposition velocity for each mode. This model allows us to calculate particle dry deposition velocities as a function of particle size, density, surface properties, and micro-meteorological conditions near the surface. Sensitivity tests and comparison with the few published measurements showed that the parameterization performed by Nho-Kim et al. [59] can predict reasonable deposition velocities for a wide particle size range over different surface types, including snow and ice-covered sea. Dry deposition velocities of 0.1, 1.4, and 2.9 cm s^−1^ were used for PHg_0.1–1.0_, PHg_1.0–2.5_ and PHg_2.5–10.0_, respectively. These deposition velocities were calculated as in Wang et al., [60] by the global multi-scale Chemistry and Transport Model MOCAGE over moderately rough surfaces (no snow or ice covered the study site) under relatively mild friction velocity conditions [59]. The uncertainty of ± 10% was applied, since the use of cascade impactor sampler could lead to possible artifacts (see above in Section 2.2).

### 3.7. Air Mass Origin

In order to characterize the transport pathways of air masses before they arrive at the study site, five-day backward trajectories every three hours (from 00 to 21 UTC), were computed using the Hybrid Single Particle Lagrangian Integrated Trajectories (HYSPLIT) model developed by NOAA and Australia’s Bureau of Meteorology [77], for the period spanning from 10 November 2017 to 13 January 2018 when aerosols samples were collected. The back-trajectories (TJ) were calculated for six arrival heights: 500 m, 1000 m, 1500 m, 2000 m, 3000 m, 4000 m, and 6000 m a.g.l. (above the ground level). HYSPLIT was initialized with the National Weather Service’s National Centers for Environmental Prediction (NCEP) Global data Assimilation System (GDAS) model data with a regular grid of 0.5° × 0.5° [78]. Errors in TJ calculations after three days are estimated in the range 10–20% of the travel distance [79], although meteorological data with coarser spatial resolution are used.

Following Mezgec et al. [80] and Caiazzo et al. [81], the whole domain is divided into a cells grid of 4° × 1° (long/lat) and number of points which each TJ spends within each cell was calculated. In order to consider only TJ able to load and transport aerosols, a threshold was applied counting TJ that spent almost 20% of the time (1 day) at heights between 100 and 1000 m. In Figure 5, only TJ at heights 500 m, 1000 m, 1500 m, 2000 m were reported since most directly influencing particulate aerosol distribution during the study period. The same was calculated for November, December, and January to highlight differences in transport and provenience as summer progressed. The threshold at 20% is arbitrary, but it is assessed that changing its value of ± 10% not lead substantial differences in results presented in Figure 5.

## 4. Conclusions

During November 2017 and January 2018, size-resolved PHg distributions were measured for the first time at Terra Nova Bay. The average PHg concentrations in PM10 was 51 ± 27 pg m^−3^ with a range of 30–90 pg m^−3^. These results were comparable to those recorded in the same sampling site and in other Antarctic areas. They also confirmed the surprisingly high concentrations of PHg characterizing the continent with respect to those measured in urban and industrialized areas. 

An inversion procedure applied to the whole dataset revealed that the size distribution of PHg in PM10 was trimodal with three peaks in the range 0.1–1.0 µm, 1.0–2.5 µm, and 2.5–10.0 µm. More than half of the particulate mercury was distributed in particle size > 1.0 µm, while PHg in the accumulation mode contributed to a less extent to the total mercury content; the percentages varying from ~3% to ~30%.

A general decrease of total PHg was observed, with the maximum value at the beginning of summer. The high concentrations of PHg in that period were probably related to the adsorption of oxidized Hg species on coarse particles, as these Hg species were either transported by air masses from the Antarctic plateau or produced locally by sea ice edges. On the contrary, the sea spray aerosol seemed to be the main source of PHg in the accumulation mode, which showed an increase during summer. When the pack ice melted, gaseous and/or oxidized Hg species were probably subjected to a gas–particle partitioning with the methanesulfonate and the not-sea-salt sulphate released from the sea surface. 

The five-day air mass back trajectories also suggested a potential contribution of the long-range transport of fine particles from anthropized areas to the PHg size distribution at Terra Nova Bay.

The opposite correlation of PHg_0.1–1.0_ and PHg_1.0–2.5_ with temperature seemed confirmed the different formation mechanisms of the two fractions. The high negative, even if not significant, correlation of PHg_1.0–2.5_ with relative humidity could explain the shift in the two coarse fractions contribution to total PHg. In fact, it could be possible that a water condensation on the first coarse mode particles occurred, causing them to grow in size as RH slightly rose during summer.

The estimated average dry deposition for PHg was 85 ngm^−2^ d^−1^ with PHg_2.5–10.0_ accounting for more than 60% of the total deposition flux. Size-resolved deposition fluxes varied following the trend of PHg concentrations in the three size fractions.

Our study provided important information for assessing the size distribution of particulate mercury in Antarctica and enhanced the general understanding of the Hg cycle in remote areas.

Nevertheless, further studies, especially on the chemical composition of atmospheric aerosol, are necessary in order to improve our understanding of Hg behavior in polar environments. The better comprehension of the particulate mercury distribution and formation mechanisms will lead to improved global transport and deposition models and could help refine pollution-control strategies around the world.

## Figures and Tables

**Table 1:** Atmospheric particulate mercury concentrations referred both to actual and standard air at Faraglione Camp during the austral summer 2017–2018. Concentrations are reported as sum of different size-segregated PM10 fractions.

Sampling Period	Actual Air Volume (m^3^)	Standard Air Volume ^b^ (m^3^)	PHg Atmospheric Concentration (pg m^−3^) ^a^	
			Actual Air	Standard Air
10/11/2017–20/11/2017	16,354	16,070	94 ± 8	95 ± 8
20/11/2017–30/11/2017	16,498	15,857	90 ± 6	94 ± 7
30/11/2017–10/12/2017	16,793	16,172	79 ± 6	82 ± 6
10/12/2014–20/12/2017	16,479	15,920	39 ± 3	40 ± 3
20/12/2017–28/12/2017	13,206	12,806	31 ± 2	32 ± 2
28/12/2017–05/01/2018	13,401	12,921	30 ± 2	31 ± 2
05/01/2018–13/01/2018	13,070	12,584	31 ± 1	32 ± 2

^a^ ± SD computed as the square root of the sum of variances. ^b^ 298 K, 1013 hPa.

**Table 2:** Summary of atmospheric particulate mercury measurements performed at various Antarctic locations and in other areas worldwide. NA: data not available.

Study Sites	Period	Mercury Concentrations Mean (Min–Max), pg m^−3^	References
Terra Nova Bay	2017–2018	51 ± 27 (30–90)	This work
Terra Nova Bay	1999–2001	12 ± 6 (~ 4–20)	[21]
Neumayer station	2000–2001	NA (15–120)	[25]
South Pole	Nov 2000	224 ± 119 (71–660)	[26]
	Dec 2001	166 ± 147 (11–827)	
McMurdo	Oct–Nov 2003	49 ± 36 (5–182)	[23]
Sweden, EU	1998–2004	8.1 ± 4.9 (1.7–19)	[2,31,32]
Germany, EU	1998–2004	42 ± 29 (21–99)	[2,31,32]
France, EU	1998–2004	108 ± 246 (1.0–662)	[2,31,32]
Italy, EU	1998–2004	15 ± 14 (1.0–46)	[32]
Spain, EU	1998–2004	30 ± 26 (9.1–86)	[32]
Barrow, Alaska	1999–2004	24 ± 25 (3–116)	[23]
Alert, Canadian Arctic	1995 to 2009	70 ± 65 (NA)	[12]
North America ruraland background areas	1995 to 2004	9.2 ± 15 (1.6–42)	[30,33,34,35]
Toronto, Canada	2003–2004	NA (14.2–39.2)	[32]
Chicago, USA		70 ± 67 (NA)	[36]
Detroit, USA	2004	NA (1.0–1345)	[37]
Mexico City, Mexico	2006	187 ± 300 (NA)	[38]
Shangai, China	2017	318 ± 144 (99–611)	[39]
Tokyo, Japan		98 ± 51 (NA)	[40]
Seoul, Korea	2010	6.8 ± 6.5 (1.1–18.5)	[29]
Global		111 ± 99 (0.6–1180)	[30]

**Table 3:** Correlation matrix (Pearson’s coefficients) among PHg size fractions and meteorological variables (temperature, T; atmospheric pressure, P; relative humidity, RH and wind speed, Wind). Significant correlation (*p* ≤ 0.05) are shown in boldface.

PHg_tot_	1.00	PHg_2.5–10.0_						
PHg_10.0–2.5_	**0.03**	1.00	PHg_1.0–2.5_					
PHg_2.5–1.0_	**0.94**	−0.28	1.00	PHg_0.1–1.0_				
PHg_1.0–0.1_	−0.70	0.17	**−0.78**	1.00	T			
T	**−0.81**	0.33	**−0.87**	**0.83**	1.00	P		
P	**−0.96**	0.18	**−0.98**	**0.80**	**0.92**	1.00	RU	
RH	−0.60	0.44	−0.73	0.47	0.61	0.71	1.00	Wind
Wind	0.30	−0.54	0.52	−0.53	−0.29	−0.38	−0.63	1.00

**Table 4:** Dry deposition fluxes of particulate mercury at Terra Nova Bay during summer 2017–2018. The uncertainty of ±10% was considered for the dry deposition fluxes reported.

Sampling Period	Dry Deposition Flux (ng m^−2^ d^−1^)			
	0.1–1.0 µm	1.0–2.5 µm	2.5–10.0 µm	Total
10/11/2017–20/11/2017	0.12	111	0	111
20/11/2017–30/11/2017	0.19	104	28	132
30/11/2017–10/12/2017	0.34	48	103	151
10/12/2014–20/12/2017	0.22	30	33	63
20/12/2017–28/12/2017	0.41	12	33	45
28/12/2017–05/01/2018	0.55	11	34	48
05/01/2018–13/01/2018	0.71	13	28	42
